# Single-cell RNAseq of Angiotensin II-induced abdominal aortic tissue identifies aneurysm-associated cell clusters in C57BL/6J mice

**DOI:** 10.1042/BSR20241235

**Published:** 2025-05-28

**Authors:** Huimin Li, Xueyu Hao, Peng Zhang, Jun Guo, Wei Li

**Affiliations:** 1 Department of Genetics and Reproductive Medicine, Shunyi Maternal and Children’s Hospital of Beijing Children’s Hospital, Beijing 101300, China; 2 Beijing Key Laboratory for Genetics of Birth Defects, Beijing Pediatric Research Institute, Beijing Children's Hospital, Capital Medical University, Beijing 100045, China; 3 MOE Key Laboratory of Major Diseases in Children, Beijing Children's Hospital, Capital Medical University, Beijing 100045, China; 4 Genetics and Birth Defects Control Center, National Center for Children’s Health, Beijing 100045, China

**Keywords:** abdominal aortic aneurysm, Angiotensin II, cell type, disease mechanism, single-cell RNA sequencing

## Abstract

Abdominal aortic aneurysms (AAAs) are life-threatening due to the rupture of aorta. Different vascular cell types are known to be involved in AAA development. However, whether any specific cell cluster plays a critical role during AAA formation is unknown. Angiotensin II (Ang II) infused mouse AAA models are commonly used to study the development and progression of AAA. We here investigate the incidence of AAA at different ages or different doses of Ang II in C57BL/6J mice. There was no AAA formation at a concentration of 1.44 mg/kg/day or 2.16 mg/kg/day at the age of 14 weeks. At the age of 20 weeks and 32 weeks, the incidence of AAA was 18.2% (6/21) and 57.1% (4/7), respectively, with a concentration of 1.44 mg/kg/day. Using single-cell RNA sequencing, we found that increased clusters of monocytes and neutrophils, macrophages, T cells, and B cells were the typical changes in AAA. A special cluster transformed from endothelial cells (malignant ECs) was identified, in which genesinvolved in lipid metabolism, including *Cd36, Lpl, Gpihbp1, Fabp4,* and *Pparg,* were highly expressed. Mice receiving Ang II treatment without AAA development showed increased fibroblasts, which may prevent the occurrence of AAA. Through cell–cell interaction analysis, we found that the Cxcl12–Cxcr4/Ackr3 axis, which functions in inflammatory ligand– receptor binding, may play a role in AAA formation. Our results reveal that specific cell clusters may contribute to the progression or prevention of AAA formation. These findings provide new clues for the pathogenesis and intervention of AAA.

## Introduction

Aortic aneurysm (AA) ranks as the 13th leading cause of death in the United States [1,2]. It has been reported that abdominal aortic aneurysm (AAA) causes approximately 0.35% of all deaths in the United States, which is mainly in individuals older than 55 years old with the male/female ratio of 1.40 [3]. AAA is usually asymptomatic until a sudden rupture. Due to the challenge of obtaining pathological specimens of human patients, animal models are crucial tools for studying the pathogenesis and progression of diseases. Angiotensin II (Ang II) infusion in mice has become the most frequently used method to induce AAA to study the molecular mechanisms and potential therapeutic strategies [4,5]. At the very beginning, it was reported that Ang II can only induce AAA in apoE^-/-^ mice, presumably the presence of hyperlipidemia was required because infusion of Ang II into apoE^+/+^ mice failed to generate aneurysm [6]. However, later studies have shown that after 6 or 10 days of Ang II infusion into 7- to 12-month-old C57BL/6 J mice at a dose of 2500 ng/kg/minute, aortic hematomas/dissections were observed in 35% (8 of 23) mice [7]. Although Ang II can induce AAA in normolipidemic mice at an older age, the incidence was significantly lower compared with that in apoE^-/-^ mice, which was 50% (8 of 16 mice) at the age of 12 weeks [8].

The incidence of AAA in C57BL/6 J mice treated with Ang II infusion varies widely among different studies [9,10]. Considering the low incidence of AAA in Ang II-treated C57BL/6 J mice, one study pointed out that a combination of Ang II and β-aminopropionitrile monofumarate (BAPN), an inhibitor of lysyl oxidase, can induce both thoracic and AAAs with a high incidence [11]. The loss of elastic fibers is an early event during aneurysm formation. The adventitious tissue, in which collagen and fibroblasts are rich, is responsible for the resistance of aneurysm in the absence of medial elastin [12]. BAPN can break the crosslinks of collagen fibers, leading to a high incidence of aneurysm formation.

The disruption of elastic fibers, degradation of collagen fibers, and destruction of vascular smooth muscle cells (SMCs) are hallmarks of AAA pathogenesis. Compelling evidence suggests that endothelial dysfunction is an early pathological episode during AAA formation, contributing to inflammation in the degenerating arterial wall [13]. However, the exact function of endothelial cells (ECs) in the development of AAA is largely unknown. Here, by using single-cell RNA sequencing (scRNAseq), we discovered a distinct cluster of heterogeneous EC subpopulations, referred to as malignant EC, showing elevated expression of genes in lipid metabolism, such as *Cd36*, *Lpl*, *Gpihbp1*, and *Fabp4*. We also found that ECs, SMCs, and fibroblasts were significantly decreased in the AAA group, whereas immune cells were notably increased. Furthermore, our data suggest that the Cxcl12–Cxcr4/Ackr3 axis functions in cell–cell interactions, which may promote inflammatory cell infiltration. These new findings reveal the key features in AAA formation, which are important for intervening AAA.

## Materials and methods

### Mouse AAA induced by Ang II infusion

In this study, C57BL/6J male mice were used. Male mice aged 14, 20, or 32 weeks were selected for pump implantation. All mice were maintained under the specific pathogen-free facility of Institute of Genetics and Developmental Biology (IGDB), Chinese Academy of Sciences (CAS). ALZET osmotic mini-pumps (Model 1004, Durect Corporation, U.S.A.) were placed subcutaneously in mice to deliver Ang II at a concentration of 1.44 mg/kg/day or 2.16 mg/kg/day, as described previously [14,15]. Pumps filled with PBS were used as normal controls (NCs) for age-matched littermates of mice. Mice were anesthetized with 2% pentobarbital sodium (45 mg/kg), and pumps were implanted through a small incision in the back of the neck, then the incision was sutured with 5–0 suture thread. All incision sites healed within seven days. All experiments were conducted in accordance with the guidelines of the Animal Care and Use Committee of IGDB, CAS.

### Aortic ultrasonography monitoring and measurement of aortic diameters

After 28 days of Ang II infusion, mice were anesthetized with 1% isoflurane. Their thorax and abdomen were depilated and coated with a developer solution. The Vevo 2100 Imaging System (FUJIFILM VisualSonics, Canada) was utilized, equipped with a 30 MHz transducer, to conduct ultrasound scans in B-mode. These scans included the longitudinal images of the ascending aorta, aortic arch, abdominal aorta, and measurements of internal diameter of the aorta. The branch of the cephalic trunk and approximately 1 cm above the branch of the coeliac trunk were chosen as the anatomical landmarks for measuring the diameter of aortic arch and abdominal aorta, respectively. AAA could be diagnosed when the ratio of the diameter of the suprarenal aorta and the non-dilated adjacent aorta is ≥1.5 [2].

### Immunohistochemical analysis and immunofluorescence staining of aortic tissues

Mice were euthanized following an intraperitoneal injection of excessive 2% pentobarbital sodium after monitoring aortic ultrasonography. The aorta was perfused through the heart with cold PBS, and segments of the aorta from the aortic root to the suprarenal abdominal aorta were isolated, with the surrounding connective tissue stripped off under a stereoscope. Mouse aortic tissue was fixed in 4% paraformaldehyde. The adventitial tissue was carefully removed, and the tissue was placed on a black gel plate for imaging of the aorta. Aortic tissue was embedded in paraffin and then sectioned. Aortic sections (5 μm) were stained with Masson’s trichrome, hematoxylin and eosin (H&E), and elastic staining reagents, respectively. Images were captured by the Nikon microscope Eclipse H550S (Nikon, Japan).

In order to confirm the cell cluster changes, the first antibodies used for immunofluorescence staining, including mouse anti-osteopontin (OPN, also named as SPP1) antibody (1:200, AF808, R&D system, U.S.A.), rabbit anti-CD68 antibody (1:100, 97778, Cell Signaling Technology, U.S.A.), mouse anti-lipoprotein lipase (LPL) antibody (1:200, ab93898, Abcam, U.K.), rabbit anti-GPIHBP1 antibody (1:20, NB110-41539, Novus Biologicals, U.S.A.), rabbit anti-CXCR4 antibody (1:100, T55380S, ABmart, China), and rabbit anti-ACKR3 antibody (1:100, A12712, ABclonal, China). The sections were fixed with 4% paraformaldehyde for 10 min and permeabilized with 1% Triton X-100 for 15 min. Then, the sections were blocked with 1% bovine serum albumin for 30 minutes and incubated with the aforementioned primary antibodies overnight at 4°C. After washing with PBS containing 0.1% (v/v) Tween 20 three times, sections were incubated with Alexa Fluor 488-conjugated goat anti-rabbit IgG (H + L) secondary antibody (A-32731, Invitrogen, U.S.A., 1:2,000) or Alexa Fluor 594-conjugated donkey anti-goat IgG (H + L) secondary antibody (A-32758, Invitrogen, U.S.A., 1:2,000) or Alexa Fluor 594-conjugated donkey anti-mouse IgG secondary antibody (A-21203, Invitrogen, U.S.A., 1:2000 dilution) or Alexa Fluor 594-conjugated goat anti-rabbit IgG (H + L) secondary antibody (A-11012, Invitrogen, U.S.A., 1:2000 dilution) for 60 min at 37°C in the dark. Finally, the sections were mounted with ProLong Gold Antifade reagent containing DAPI (Invitrogen, U.S.A.). Images were captured using the Zeiss LSM 880 Confocal Microscope (Zeiss, Germany).

### scRNAseq and data analysis

We conducted scRNAseq on three groups of mice: 20-week-old mice receiving Ang II (1.44 mg/kg/day) or PBS infusion. The mice receiving Ang II were further divided into two groups (based on ultrasonography monitoring, with or without AAA). After conducting aortic ultrasonography monitoring, the mice were killed. The aortas were perfused through the heart with cold PBS, and the abdominal aortas were isolated and pooled from four mice in each group for single-cell suspension. The adventitial tissue was carefully removed, and the abdominal aortas were washed three times with cold PBS, then transferred to a 2 ml centrifuge tube containing 500 µl RPMI 1640 (11875093, Thermo Fisher Scientific, U.S.A.). The tissue was cut with scissors as small as possible. 1 ml RPMI 1640 containing 0.125% trypsin (25200056, Invitrogen, U.S.A.) was added to the tube, which was then placed on ice and gently mixed. The tubes were incubated at 37°C with slow rotation for 30 min. Following this, the tubes were centrifuged at 4°C, 300 g for 5 min, completely discard the supernatant, and 1.5 ml PB (BSA in PBS, final concentration is 0.5%) buffer was added to resuspend the tissue. The supernatant was discarded again as described above. 1.5 ml RPMI 1640 containing 1 mg/ml collagenase II (1148090, Sigma-Aldrich, Germany) was added into the centrifuge tube, and the tubes were rotated slowly at 37°C for 30 min. The samples were filtered with a 70 µM filter. The strainer and 2 ml centrifuge tube were washed with RPMI 1640, respectively, and the final volume was 10 ml. The samples were centrifuged at 4°C, 300 g for 5 min, then completely discarded the supernatant, and the cells were resuspended with 400 µl PB buffer. Acridine Orange (AO)/Propidium Iodide (PI) staining was conducted for quality inspection. After erythrocyte lysis, cell debris was removed.

Analyses were performed using R software (https://www.r-project.org/, version 3.6) and primarily utilizing the ‘Seurat’ packages. To process the data, cells were filtered. A total of 29,435 cells (before-treatment specimens) were retained based on the filtering criteria: 1) the number of genes detected per cell was >300 and <4000 and 2) the percent of mitochondrial genes was <0.10. Then, we selected the expression matrix of cells from pre-treatment samples as the input. Expression profiles were log-normalized and scaled, with the percentage of mitochondrial genes regressed out. Clusters were determined using the first 15 principal components and visualized through the uniform manifold approximation and projection (UMAP) dimensional reduction method for each sample. The identified cell clusters could be readily assigned to known cell lineages through conventional marker genes. The cell–cell interactions and receptor–ligand pairs between all major cell types were estimated using CellPhoneDB (version 2.1.2) (www.cellphonedb.org). Potential interactions between the two cell types were inferred through gene expression levels using 1000 permutation tests. Then, the resulting adjacency matrices were generated for all cell–cell interactions and visualized on heatmaps. Cell–cell interactions within identical cellular lineages were excluded. Only gene pairs involved in receptor-ligand interactions in cell types of interest were visualized if the combined *P* value was <0.001 (calculated by multiplying all *P* values within each gene pair).

### Statistical analysis

Data were presented as mean ± SEM. All statistical tests were two-sided, a significant difference was considered when the *P* value was <0.05. Comparisons of the aortic diameters were analyzed using the student’s *t*-test and one-way ANOVA. Comparisons of the AAA incidence rates were analyzed using Fisher’s exact test. Comparisons of differences in the composition ratios of cell clusters were analyzed using chi-square test. GraphPad Prism 9 was used for statistical analyses. Differential gene expression testing was conducted in Seurat as outlined in the scRNAseq section.

## Results

### Ang II-induced AAA in mice is age-dependent

**Figure 1:**
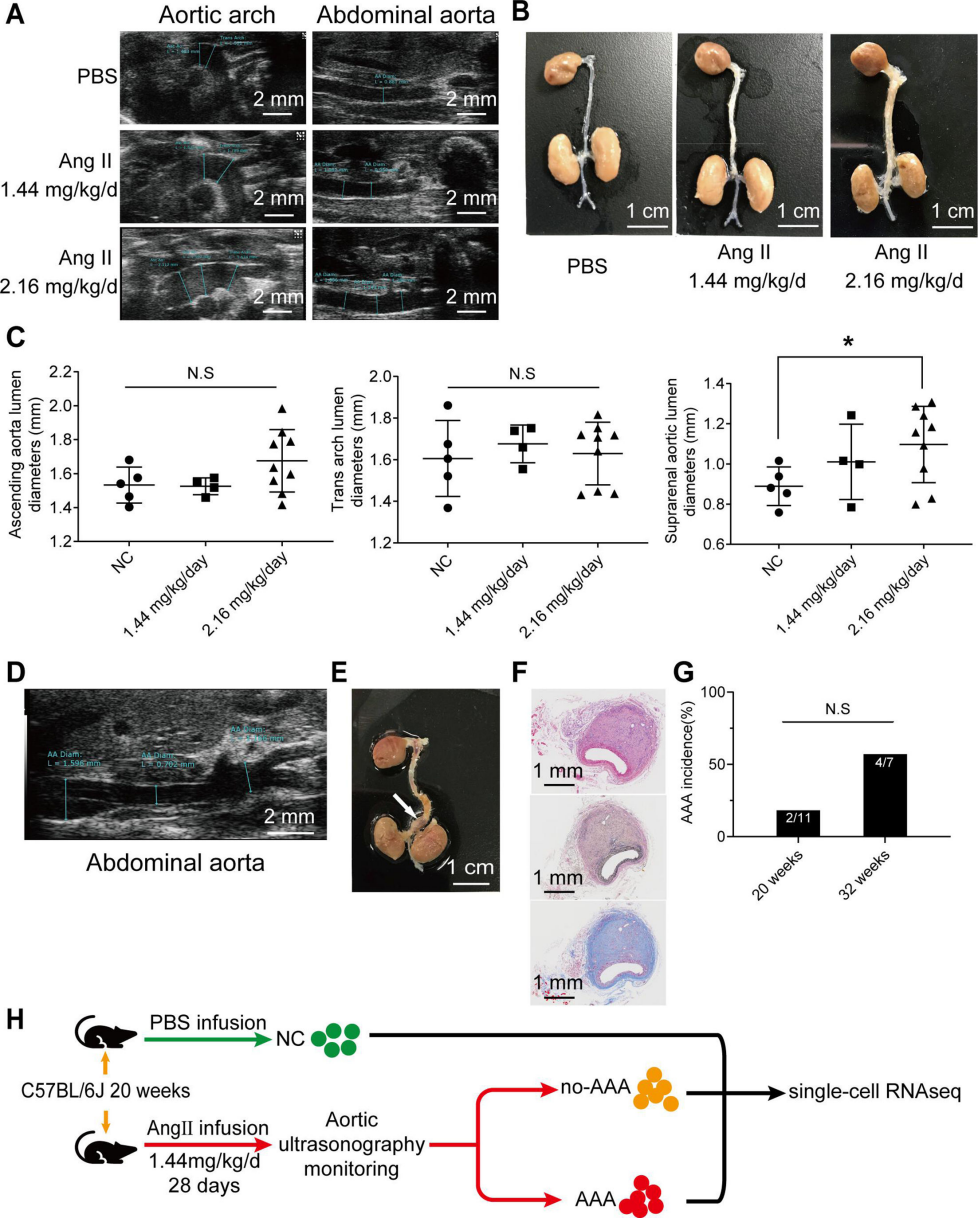
Age and dose effects in Ang II-induced AAA in mice. (**A**) Representative ultrasound images from C57BL/6 J mice treated with either PBS or Ang II (1.44 mg/kg/day or 2.16 mg/kg/day) at the age of 14 weeks. Scale bars: 2 mm. (**B**) Representative images of the mouse aorta at 14 weeks of age. C57BL/6 J mice were infused with Ang II (1.44 mg/kg/day or 2.16 mg/kg/day for 28 days). Negative control (NC) mice were C57BL/6 J mice of the same age and gender as the experimental group, infused with PBS for the same duration. Scale bars: 1 cm. (**C**) There were no significant differences in the lumen diameter of the ascending aorta, aortic arch, and abdominal aorta when comparing Ang II-treated mice (1.44 mg/kg/day, *n* = 4; 2.16 mg/kg/day, *n* = 9) with the PBS- treated mice (NC, *n* = 5) at the age of 14 weeks (ANOVA, *i>P* = 0.1427, 0.7838, and 0.1222). However, the diameters of the suprarenal abdominal aorta were slightly increased in Ang II (2.16 mg/kg/day) infused mice compared with the NC group. *P = 0.0429. The means ± SEM of the lumen diameter can be found in Table 1. N.S. not significant. (**D**) A representative ultrasound image of AAA from Ang II-treated mice (1.44 mg/kg/day) for 28 days at the age of 20 weeks. Scale bar: 2 mm. The diameter of the dilated suprarenal aorta was 1.596 mm, while the non-dilated adjacent measured 0.70 mm. The ratio of dilated to non-dilated aorta was 2.27, which exceeds the diagnostic criteria of 1.5 for AAA. (**E**) A representative image of AAA from mice treated with Ang II (1.44 mg/kg/day) for 28 days at the age of 20 weeks, as indicated by white arrow. Scale bar: 1 cm. (**F**) Representative images of AAA sections stained with hematoxylin and eosin (H&E, top), elastic staining (middle), and Masson’s trichrome (bottom). Scale bars: 1 mm. (**G**) AAA was detected in 2 out of 11 mice at the age of 20 weeks and in 4 out of 7 mice at the age of 32 weeks. N.S. not significant. (**H**) A schematic diagram of the experimental design for single-cell RNAseq. AAA, abdominal aortic aneurysm; Ang II, angiotensin II.

To investigate the impact of age and dosage on Ang II-induced AAA, we observed no AAA formation at doses of 1.44 mg/kg/day or 2.16 mg/kg/day in 14-week-old mice. However, a minor suprarenal aortic dilation was noted at the 2.16 mg/kg/day dose compared with the NC group (*P*<0.05) (Figure 1A–C, Table 1). At the age of 20 weeks and 32 weeks, both at a concentration of 1.44 mg/kg/day, the incidence of AAA was 18.2% (2/11) and 57.1% (4/7) (Figure 1D–G). Although it appears that the incidence increased with age, the difference in the occurrence of aortic dissections between these two age groups was not significant (*P*>0.05), which may be due to the relatively small number of mice used. To precisely define cell-specific transcriptomic changes associated with Ang II-induced AAA, we performed scRNAseq of isolated abdominal aortic tissues from 20-week-old mice, as illustrated in Figure 1H.

**Table 1:** The lumen diameter of ascending aorta, aortic arch and abdominal aorta which were expressed as means ± SEM.

Diameter (mm)	Negative Control (PBS infusion, *n* = 5)	Ang II Infusion (1.44 mg/kg/day, *n* = 4)	Ang II Infusion (2.16 mg/kg/day, *n* = 9)
Ascending aorta	1.533 ± 0.04750	1.525 ± 0.02466	1.675 ± 0.06142
Aortic arch	1.606 ± 0.08163	1.676 ± 0.04532	1.630 ± 0.05024
Abdominal aorta	0.8892 ± 0.04288	1.010 ± 0.09360	1.097 ± 0.06319

### Abdominal aortic tissue scRNAseq identifies aneurysm-associated cell clusters

**Figure 2:**
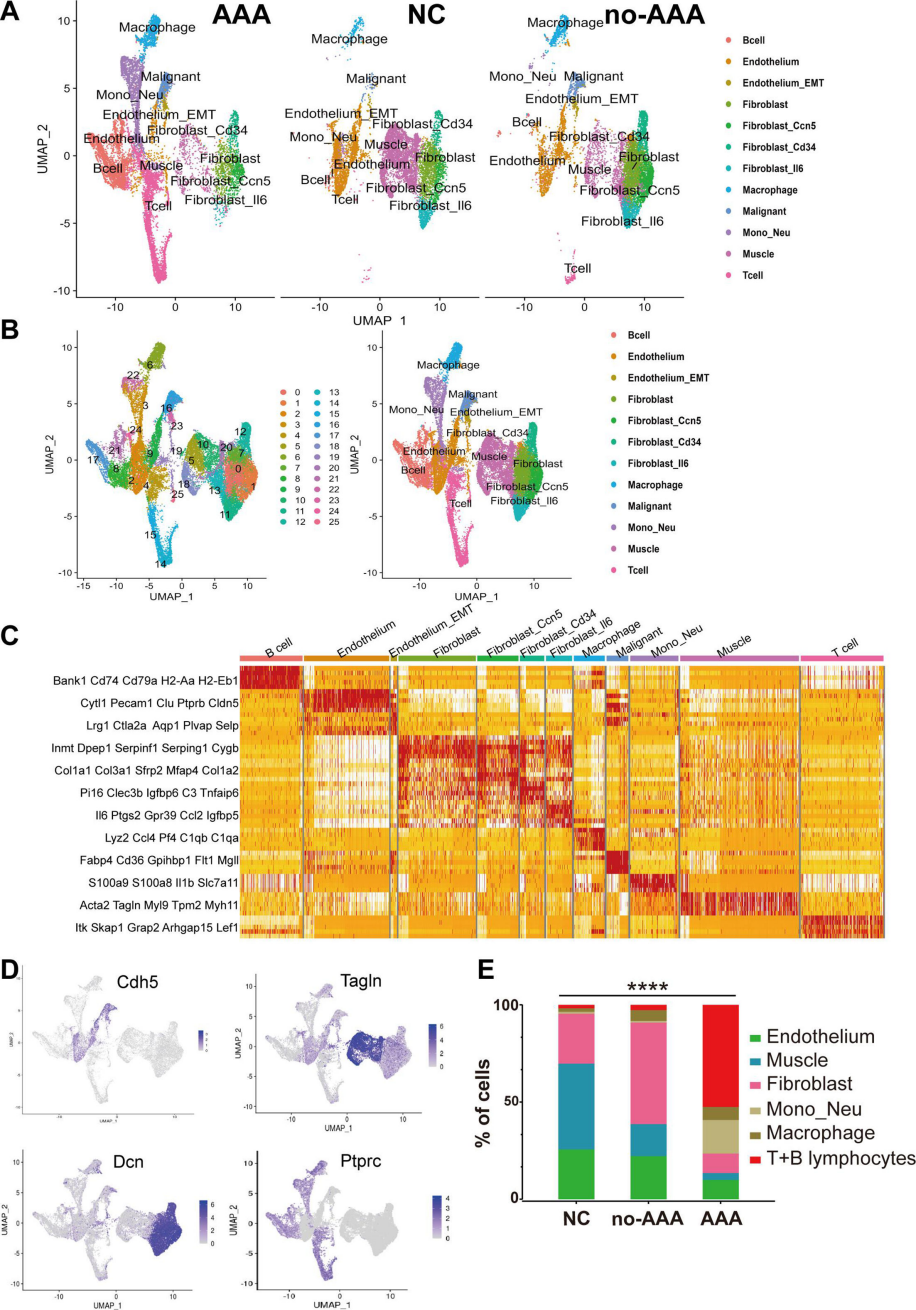
Cell types classified through single-cell RNA sequencing (scRNAseq) of mouse aortas. (**A**) After quality control, 8,716, 8,443, and 12,276 cells from the normal control (NC), without AAA formation (no-AAA), and with AAA formation (AAA) groups were selected for clustering analysis, respectively. Uniform manifold approximation and projection (UMAP) plot of aggregating cells from the NC, no-AAA, and AAA groups. (**B**) UMAP plot of cell clusters, illustrating the partition of 26 distinct clusters (upper panel) and cell identities (lower panel). The clusters listed in brackets are assigned to different cell types: endothelium (2, 9, 19, 24), endothelium_EMT (23), malignant (16), muscle (5, 10, 13, 18, 20), fibroblast (0, 7), fibroblast_Ccn5 (1), fibroblast_Cd34 (12), fibroblast_Il6 (11), monocyte_neutrophil (mono_neu; 3, 22), macrophage (6), B cell (8, 17, 21), T cell (4, 14, 15, 25). (**C**) Heat map displaying the top five genes with specific expression for each cell cluster, illustrating the relative expression levels in each cluster. The top five cluster-specific markers for each cluster were selected from all markers of each cluster based on the average log-fold change. Rows represent genes, and columns represent clusters. The width of each cluster represents its proportion in all cells of the three groups. A complete list of marker genes for each cluster can be found in Table 2. (**D**) The expression of marker genes for cell identification is displayed on the UMAP plot (gene expression log-normalized by Seurat). *Cdh5* is the marker of endothelium cells, *Tagln* is the marker of smooth muscle cells, *Dcn* is the marker of fibroblasts, *Ptprc* is the marker of immune cells. (**E**) The proportions of each identified cell type in the NC, no-AAA, and AAA groups are shown in the stacked column chart. The differences of each cell type among the three groups are statistically significant, ****P<0.0001 by Chi-square test. In Figures 2–7, the cluster of muscle represents smooth muscle cells, mono_neu represents monocytes and neutrophils.

After alignment and quality control, 8,716, 8,443, and 12,276 cells from three groups of abdominal aortas of NC, without AAA (no-AAA) and with AAA (AAA), respectively, were combined and subjected to further analysis. Unsupervised clustering using the Seurat R package revealed 26 distinct clusters, representing 12 cell lineages, including all major aortic cell types (Figure 2A–C, Table 2). In accordance with the canonical markers [16], the major clusters were ECs [Cluster 2, 9, 16, 19, 23, 24; *Cdh5*^+^ (encoding a calcium-dependent glycoproteins)], SMCs [Cluster 5, 10, 13, 18, 20; *Tagln*^+^ (encoding an SMC-specific cytoskeletal protein)], fibroblasts [Cluster 0, 1, 7, 11, 12; *Dcn*^+^ (encoding a protein that regulates collagen fibril assembly)], and immune cells [Cluster 3, 4, 6, 8, 14, 15, 17, 21, 22, 25; *Ptprc*^+^ (encoding Cd45)] (Figure 2D).

**Table 2:** A complete list of marker genes of 12 clusters.

Endothelium					Endothelium_EMT				
*Cytl1*	*Pecam1*	*Clu*	*Ptprb*	*Cldn5*	*Lrg1*	*Ctla2a*	*Aqp1*	*Plvap*	*Selp*
**Malignant**					**Muscle**				
*Fabp4*	*Cd36*	*Gpihbp1*	*Flt1*	*Mgll*	*Acta2*	*Tagln*	*Myl9*	*Tpm2*	*Myh11*
**Fibroblast**					**Fibroblast_ Il6**				
*Inmt*	*Dpep1*	*Serpinf1*	*Serping1*	*Cygb*	*Il6*	*Ptgs2*	*Gpr39*	*Ccl2*	*Igfbp5*
**Fibroblast_Cd34**					**Fibroblast_Ccn5**				
*Pi16*	*Clec3b*	*Igfbp6*	*C3*	*Tnfaip6*	*Col1a1*	*Col3a1*	*Sfrp2*	*Mfap4*	*Col1a2*
**Macrophage**					**Monocyte_Neutrophil**				
*Lyz2*	*Ccl4*	*Pf4*	*C1qb*	*C1qa*	*S100a9*	*S100a8*	*Il1b*	*Slc7a11*	
**B cell**					**T cell**				
*Bank1*	*Cd74*	*Cd79a*	*H2-Aa*	*H2-Eb1*	*Itk*	*Skap1*	*Grap2*	*Arhgap15*	*Lef1*

In the AAA tissues, the proportions of three major aortic cell types, including SMCs, fibroblasts, ECs were significantly reduced. This reduction may explain why the thickness of the aortic aneurysm wall decreased and its mechanical function was compromised. Consistent with previous findings indicating that inflammation is a fundamental element in the development of AAA [17], we observed a significant increase in immune cells such as monocytes_ neutrophils, macrophages, and T and B lymphocytes in the AAA group. Notably, T and B lymphocytes were the predominant cell populations in AAA tissues (29.9% and 22.6%, respectively). These finding suggest that inflammation in the vasculature is a hallmark of AAA. The differences in composition ratios among the three groups were statistically significant (*P*<0.0001) (Figure 2E).

Both IL-1β and TNF-α have been associated with the progressive inflammatory process that promotes aneurysm progression [18,19]. These two factors have long been considered among the most important factors promoting the inflammatory process in diseases. In our study, the expression of *Il1b* and *Tnf* was up-regulated significantly in the clusters of macrophages and mono_neu, which may promote the inflammatory process (Supplementary Figure S1A and B). IL-6 is produced by multiple cell types and mediates a diverse set of pathophysiologic functions. In our study, the expression of *Il6* was relatively high in vascular cells including four fibroblast clusters, endothelium, and muscle (Supplementary Figure S1C). *Il6* is higher in no-AAA group than in NC group, which may indicate that IL-6, as an important cytokine, was involved in the formation of AAA.

Compared with NC, the proportions of ECs and SMCs decreased slightly in the no-AAA group, while the proportion of immune cells slightly increased. In contrast, we found that the proportion of fibroblasts increased significantly in the no-AAA group, accounting for almost half of the total cells (17.6% in NC, 53.9% in no-AAA, 14.1% in AAA, *P*<0.0001). Taken together, the decreased number of three major aortic cell types and the expansion of immune cells may represent typical features in AAA.

### Distributional pattern of EC subpopulations differentiates the AAA group

**Figure 3:**
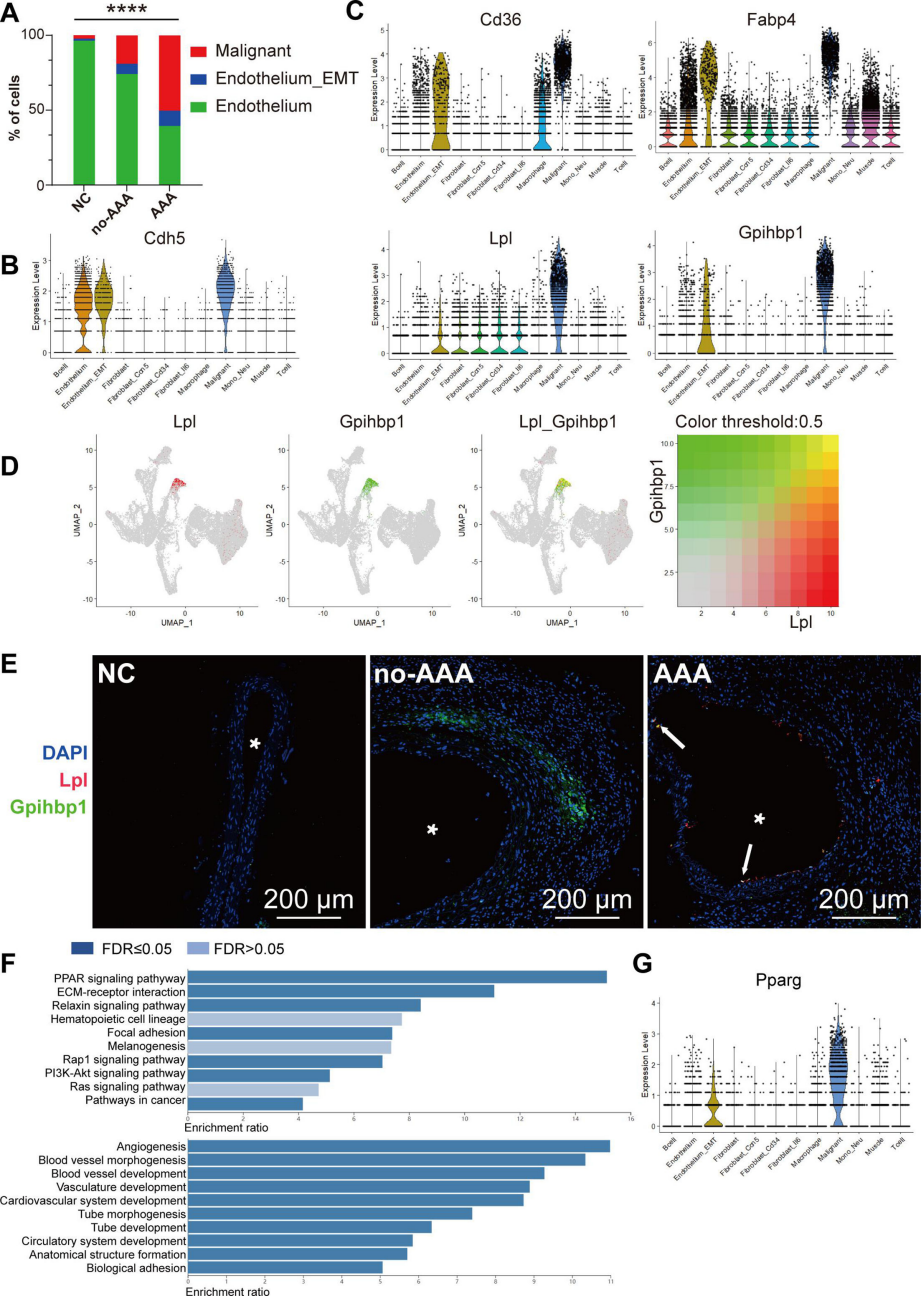
Comparison of endothelial cell subpopulations in the abdominal aorta among the NC, no-AAA, and AAA groups. (**A**) Stacked column chart shows the proportion of endothelial cell subtypes in the NC, no-AAA, and AAA groups. The proportion of endothelial cell subtypes among the three groups was statistically significant, ****P<0.0001 by Chi-square test. In the NC group, the proportions of malignant, endothelium_EMT, and endothelium were 2.2%, 1.4%, and 96.4%, respectively. In the no-AAA group, the proportions of malignant, endothelium_EMT, and endothelium were 19.0%, 6.9%, and 74.1%, respectively. In the AAA group, the proportions of malignant, endothelium_EMT, and endothelium were 50.4%, 10.2%, and 39.4%, respectively. (**B**) The expression of *Cdh5* in 12 clusters of cells from NC, no-AAA, and AAA groups is visualized by feature plots, where three subtypes of epithelial cells can be differentiated. (**C**) The expression of selected marker genes *Cd36, Fabp4, Lpl, Gpihbp1* for the malignant cluster from the NC, no-AAA, and AAA groups is visualized through feature plots. (**D**) A dual-plot gene expression heatmap shows the highest co-expression of Lpl and Gpihbp1 in the malignant cluster. (**E**) Representative immunofluorescence images of aorta sections stained with anti-Lpl (red) and anti-Gpihbp1 (green). White arrows indicate the co-expression of Lpl and Gpihbp1. Scale bars, 200 μm. The asterisk symbols indicate the location within the lumen of the abdominal aorta. (**F**) Bar chart of enrichment ratios for Kyoto Encyclopedia of Genes and Genomes (KEGG, upper panel) and Gene Ontology (GO, lower panel) pathways in the malignant cluster of 1601 diferentially expressed genes (including 1168 up-regulated genes and 433 down-regulated genes). False discovery rate (FDR) ≤0.05 indicates significantly enriched pathways, as indicated by the dark blue color. Enrichment ratio = the number of observed genes/ the number of expected genes from each GO or KEGG category in the gene list. (**G**) The expression of *Pparg* in the 12 clusters from the NC, no-AAA and AAA groups is visualized by feature plots. Note that *Pparg* is highly expressed in malignant. AAA, abdominal aortic aneurysm; EMT, epithelial-mesenchymal transition; NC, negative control.

Endothelial dysfunction plays a crucial role in the cellular transformation and inflammation of the media and adventitia [20]. In our study, ECs were classified based on cellular markers [21,22] into three groups: malignant (cluster 16, with markers *Cd36*, *Lpl*, *Gpihbp1*, and *Fabp4*), endothelium_EMT (epithelial–mesenchymal transition) (cluster 23, with markers *Lrg1*, *Ctla2a*, *Aqp1*, *Plvap*, and *Selp*), and endothelium (clusters 2, 9, 19 and 24, with markers *Cytl1*, *Pecam1*, *Clu*, *Ptprb*, and *Cldn5*). We found that the proportion of ECs significantly decreased in the AAA group, but there was no significant decrease in the no-AAA group (25.64% in NC, 22.18% in no-AAA, and 9.92% in AAA). The cluster of malignant cells mainly presented in AAA tissues (5% of all cells) and no-AAA tissues (4.22% of all cells), while occurring at a low frequency in NC tissues (0.56% of all cells). The proportion of malignant ECs increased significantly (2.17% in NC, 19.04% in no-AAA, 50.41% in AAA), while the proportion of endothelium ECs decreased significantly (96.40% in NC, 74.08% in no-AAA, and 39.41% in AAA). The differences in composition ratio of these three clusters among the three groups were statistically significant (Figure 3A). The EC marker *Cdh5* was expressed in the clusters of endothelium, endothelium_EMT, and malignant. Our results suggest that the distributional pattern of EC subpopulations in the three groups differs, allowing for differentiation between the NC, no-AAA, and AAA groups (Figure 3B).

We identified that *Cd36, Lpl, Gpihbp1*, and *Fabp4* were markers of the malignant cluster (Figure 3C). *Cd36* is extensively expressed on the surface of ECs throughout the aorta [23], where it functions as a transporter of fatty acids (FAs) [24]. EC-specific *Cd36* knockout mice showed diminished transfer of long-chain FAs across ECs [25]. *Fabp4* is a canonical PPARγ target gene involved in FA transport. Cd36 and Fabp4 are specialized transporters that facilitate the exogenous uptake of FAs from the surrounding microenvironment [26]. Our results showed the highest co-expression of *Lpl* and *Gpihbp1* (Figure 3D and E), confirming that the cells expressing *Lpl* and its partner *Gpihbp1* transport FA to the capillary lumen [27].

The 1601 differentially expressed genes (including 1168 up-regulated genes and 433 down-regulated genes) in the malignant cluster were used for functional annotation. By conducting Gene Ontology (GO) analysis, it was found that functions related to angiogenesis and blood vessel morphogenesis were up-regulated in the cluster of malignant (Figure 3F). In addition, Kyoto Encyclopedia of Genes and Genomes (KEGG) analysis revealed enrichment in the pathways of peroxisome proliferator-activated receptor (PPAR) signaling, relaxin signaling, and ECM-receptor interaction (adjusted *P* value <0.05 for all pathways) (Figure 3F). *Pparg* (which encodes PPARγ), a gene that regulates glucose and lipid metabolism, endothelial function, and inflammation, was highly expressed in the cluster of malignant (Figure 3G). Loss of PPARγ function in the vascular endothelium enhances atherosclerosis in high-fat diet-fed apoE^-/-^ mice [28]. Aortic inflammation during AAA formation is dynamic. Protective anti-inflammatory cytokines are up-regulated in the early compensatory stage. However, pro-inflammatory cytokines are dominant in the late decompensatory stage. PPARγ is likely to continue to up-regulate the expression of anti-inflammatory cytokines, extend the compensatory stage, and decelerate the process of AAA development and rupture [29]. By using the Ang II-induced murine model of aortic aneurysm, Jones et al. found that treatment with PPARγ agonist rosiglitazone had ameliorative effects on aneurysm development and rupture, which exerted a protective effect on aneurysm development and rupture [30]. PPARγ is also important in lipid metabolism as it regulates genes involved in the release, transport, and storage of FAs, including Lpl and the FA transporter Cd36 [31]. In ECs, FAs transport is facilitated by PPARγ, which stimulates the expression of *Cd36* and *Fabp4* [32]. In mice which lack PPARγ in the endothelium, the *Cd36* and *Fabp4* expression levels were decreased significantly in the endothelium, serum-free fatty acid and triglyceride (TG) levels were increased [32].

Ang II can induce AAA in normolipidemic mice at an older age, although the incidence was significantly lower compared with that in apoE^-/-^ mice. The development of AAA is age-dependent, and aging is a major risk factor for cardiovascular disease [33]. By single-cell transcriptomic analysis, Zhang et al. compared aortas and coronary arteries in young and old monkeys. They found differentially expressed genes between young and old monkeys, and the core pathways annotated for these genes are involved in ‘response to lipids’ [34]. Therefore, we speculated that advancing age accompanied with abnormal lipid metabolism may contribute to the development of AAA. The cluster of malignant EC appeared after Ang II infusion, in which *Cd36*, *Lpl*, *Gpihbp1*, *Fabp4*, and *Pparg* were highly expressed. Thus, in the cluster of malignant, we speculated that the high expression of *Cd36, Lpl, Gpihbp1, Fabp4,* and *Pparg* was important in the metabolism of FAs and may contribute to endothelial dysfunction.

### Monocyte recruitment and macrophage accumulation play an important role in aneurysm formation

**Figure 4:**
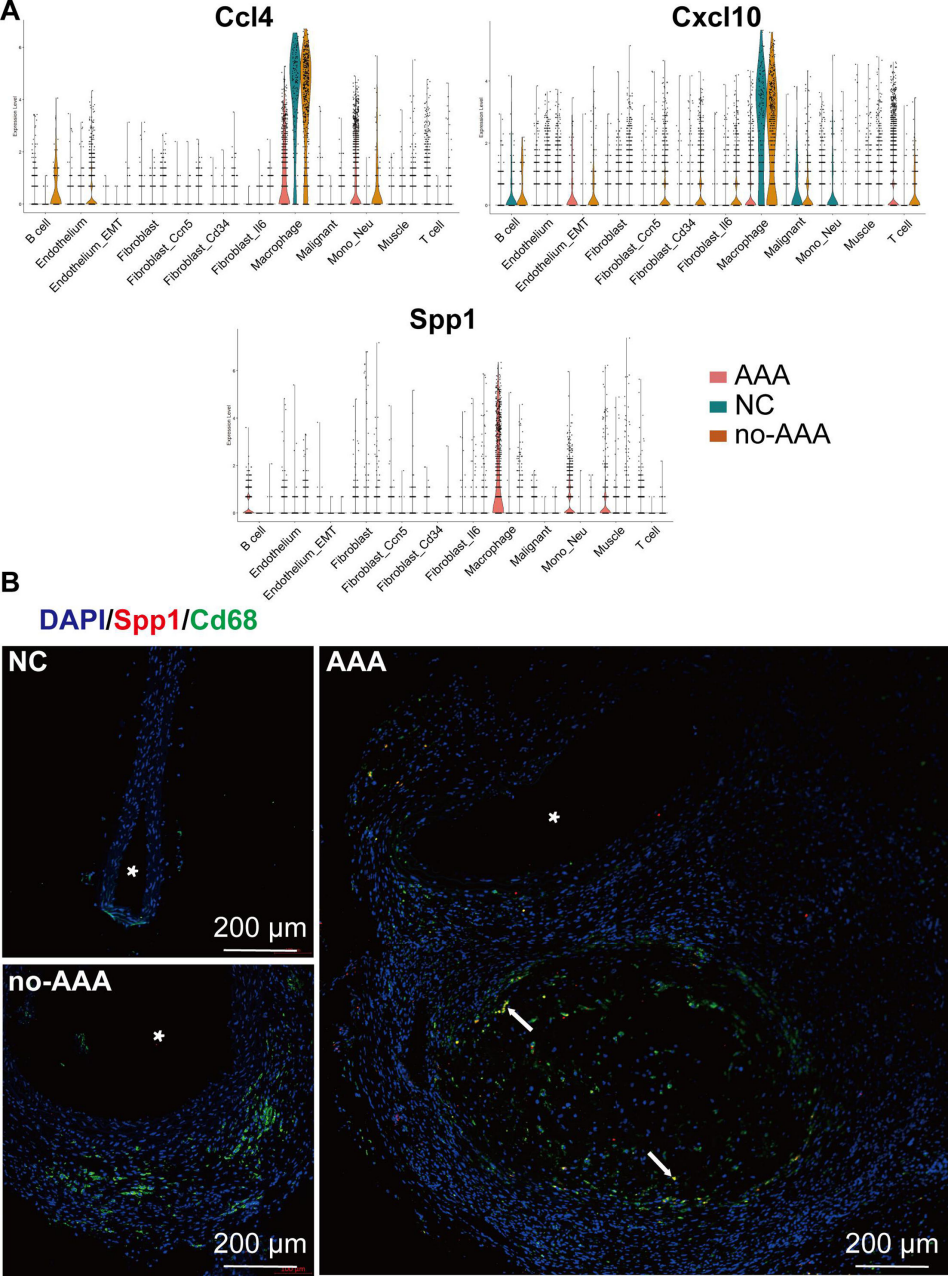
Comparison of macrophages in the abdominal aorta among the NC, no-AAA, and AAA group. (**A**) The expression of selected marker genes *Ccl4, Cxcl10, Spp1* for the macrophage cluster from NC, no-AAA, and AAA groups is visualized through feature plots. (**B**) Representative immunofluorescence images of aorta sections stained with anti-Spp1 (red) and anti-Cd68 (green). White arrows indicate the co-expression of Spp1 and Cd68. Scale bar, 200 μm. The asterisk symbols indicate the location within the lumen of the abdominal aorta. AAA, abdominal aortic aneurysm; NC, negative control.

Monocytes and macrophages play a crucial role in aneurysm formation by influencing the migration of leukocytes from the circulating bloodstream to the arterial subendothelial space. We found that the proportion of the mono_neu cluster (representing monocytes and neutrophils) significantly increased in the AAA group (17.24%) compared with 1.05% and 0.87% in the NC group and no-AAA group, respectively. Compared with monocytes, there was no significant difference in the proportion of macrophages between AAA and no-AAA, which were 6.76% and 5.60%, respectively. It has been reported that *Spp1*-deficient leukocytes provide substantial protection against Ang II-accelerated atherosclerosis and AAA formation [35]. In our study, compared with macrophages in NC and no-AAA, we identified a subpopulation of macrophages in AAA that exhibited significantly up-regulated expression of *Spp1* and notably down-regulated expression of *Ccl4* and *Cxcl10* (Figure 4A). This indicates that these cells may have a crucial role in AAA pathogenesis. In order to validate the existence and localization of Spp1, our results showed that Spp1 was present in the AAA tissues and colocalized with Cd68 through co-immunofluorescence staining. In contrast, Spp1 was not observed in the NC and no-AAA groups (Figure 4B).

### Fibroblast expansion may prevent the development of aneurysm upon Ang II treatment

**Figure 5:**
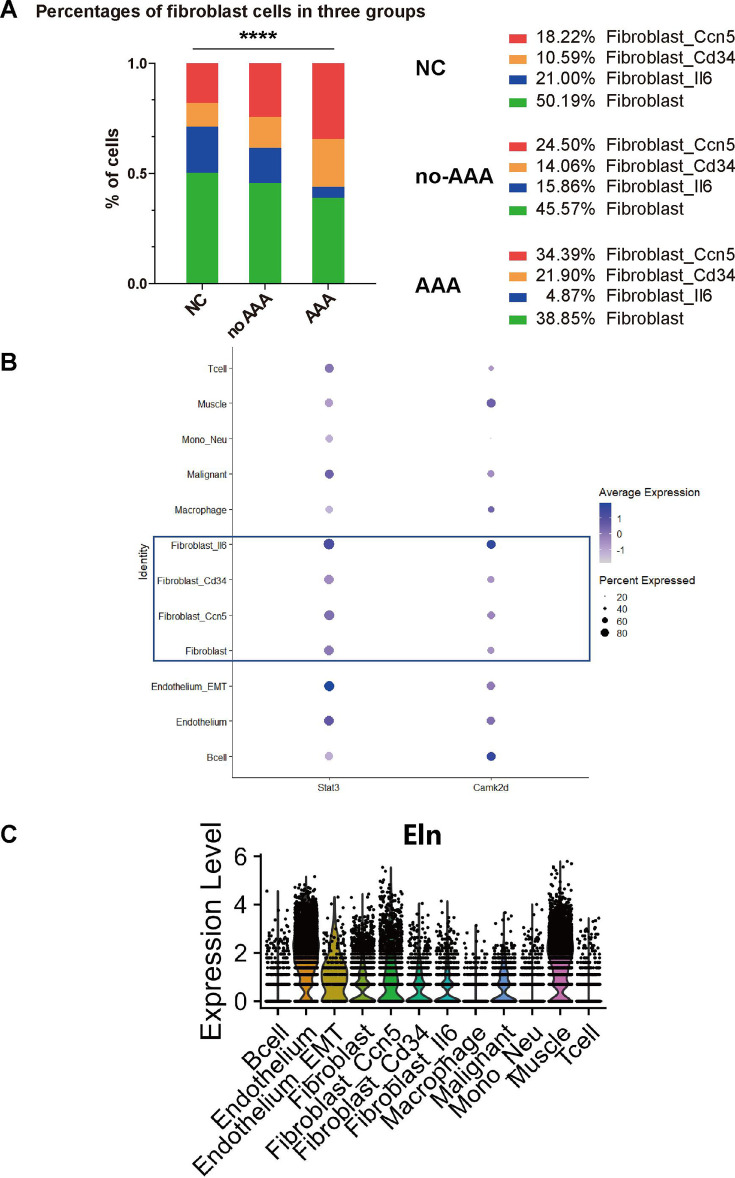
Comparison of fibroblast subpopulations in the abdominal aorta among the NC, no-AAA, and AAA group. (**A**) Stacked column chart depicting the proportion of fibroblast subtypes in NC, no-AAA, and AAA groups. The proportions of fibroblast subtypes in the three groups listed are statistically significant, ****P<0.0001 by Chi-square test. (**B**) The expression of *Camk2d* and *Stat3* was up-regulated in the cluster of fibroblast_Il6. The color key from gray-violet to blue indicates low to high expression levels. The dot size indicates the percentage of cells expressing the two indicated genes. (**C**) The expression of elastin (*Eln*) in different cell clusters. AAA, abdominal aortic aneurysm; NC, negative control.

We next explored why about two thirds of mice receiving Ang II infusion did not develop AAA. Fibroblasts were identified as the largest cell population in no-AAA tissues. These fibroblasts have been differentiated into four major subpopulations: fibroblast, fibroblast_Il6, fibroblast_Ccn5, and fibroblast_Cd34 [36,37]. Il-6 is a member of the interleukin family. Camk2d (Ca^2+^/calmodulin-dependent protein kinase II) is known to play an important role during cardiac hypertrophy [38]. Signal transducer and activator of transcription 3 (Stat3) is a member of the STAT protein family, which acts as a transcription activator, and response to cytokines and growth factors. Il-6 can directly activate Camk2d, the latter then activate Stat3 [39]. The Il6–Camk2d–Stat3 pathway is involved in fibroblast activation, leading to cardiac fibrosis [40]. In the cluster of *Il6*^+^ fibroblasts, the expression of *Camk2d* and *Stat3* was up-regulated (Figure 5A), suggesting that this cluster may promote vascular fibrosis.

Cd34 is one of the markers for myofibroblast (MF). MFs are specialized cells that originate in pathophysiological conditions and contribute to tissue repair during wound healing [41]. Cellular communication network 5 (Ccn5), which is known as a secreted protein, mediates anti-fibrotic activity by inhibiting fibroblast-to-myofibroblast transition [42,43]. In consideration of the increased proportions of both *Cd34*^+^ fibroblasts and *Ccn5*^+^ fibroblasts in no-AAA compared with NC, we speculate that under the stimulation of Ang II, *Cd34*^+^ fibroblasts proliferated, which may contribute to tissue repair. Additionally, *Ccn5*^+^ fibroblasts increased to balance the increase of *Cd34*^+^ fibroblasts due to their anti-fibrotic activity [42,43]. Our data showed that although the total number of fibroblasts differed significantly between no-AAA and AAA tissues (52.21% vs 10.04%, *P*<0.0001), the changes in fibroblast proportions exhibited a similar trend in these two groups when compared with NC. The cell proportions of fibroblast and fibroblast_Il6 were lower in no-AAA and AAA tissues compared with the NC group, while the cell proportions of fibroblast_Cd34 and fibroblast_Ccn5 were higher in no-AAA and AAA tissues compared with NC (Figure 5B). The differences in composition ratios of these four clusters among the three groups were statistically significant (*P*<0.0001). Thus, changes in fibroblast subtypes may be associated with AAA development.

The loss of elastic fibers is an early event during aneurysm formation. The adventitious tissue, in which collagen and fibroblasts are rich, is responsible for the resistance of aneurysm in the absence of medial elastin [12]. In our study, the expression of *Eln* was high in the cluster of endothelium, fibroblast and fibroblast_Ccn5, and muscle, and the proportions of these clusters were reduced significantly in the AAA group (Figure 5C).

### The Cxcl12–Cxcr4/Ackr3 axis may function in AAA development

**Figure 6:**
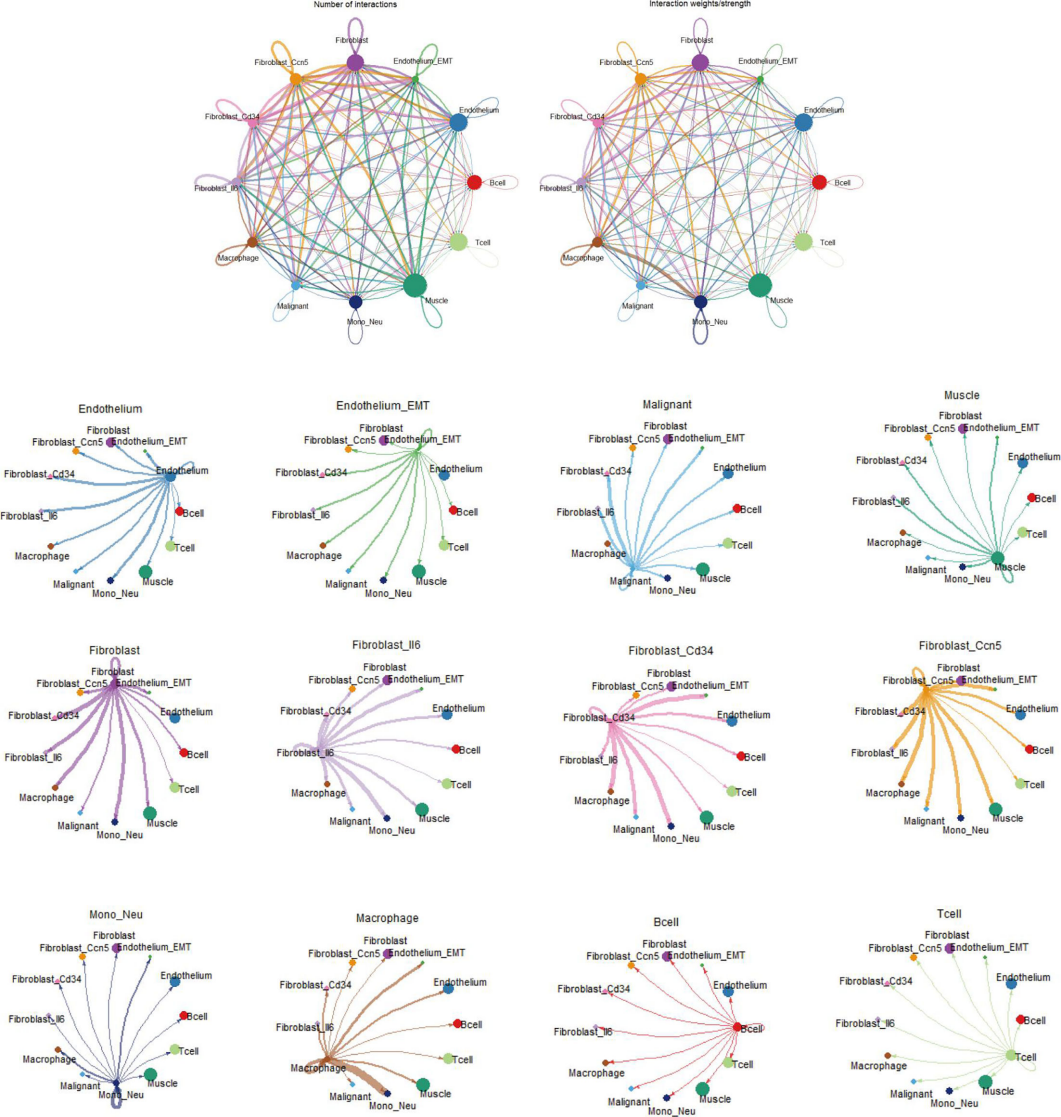
Visualization and analysis of cell–cell communication through circle plots. The number and strength of interactions between pairs of 12 cell clusters are shown. The sizes of the circles are proportional to the number of cells in each cluster; the larger the circle, the greater the number of cells. The width of the edges represents the communication probability; the thicker the line, the more likelihood of communication.

**Figure 7:**
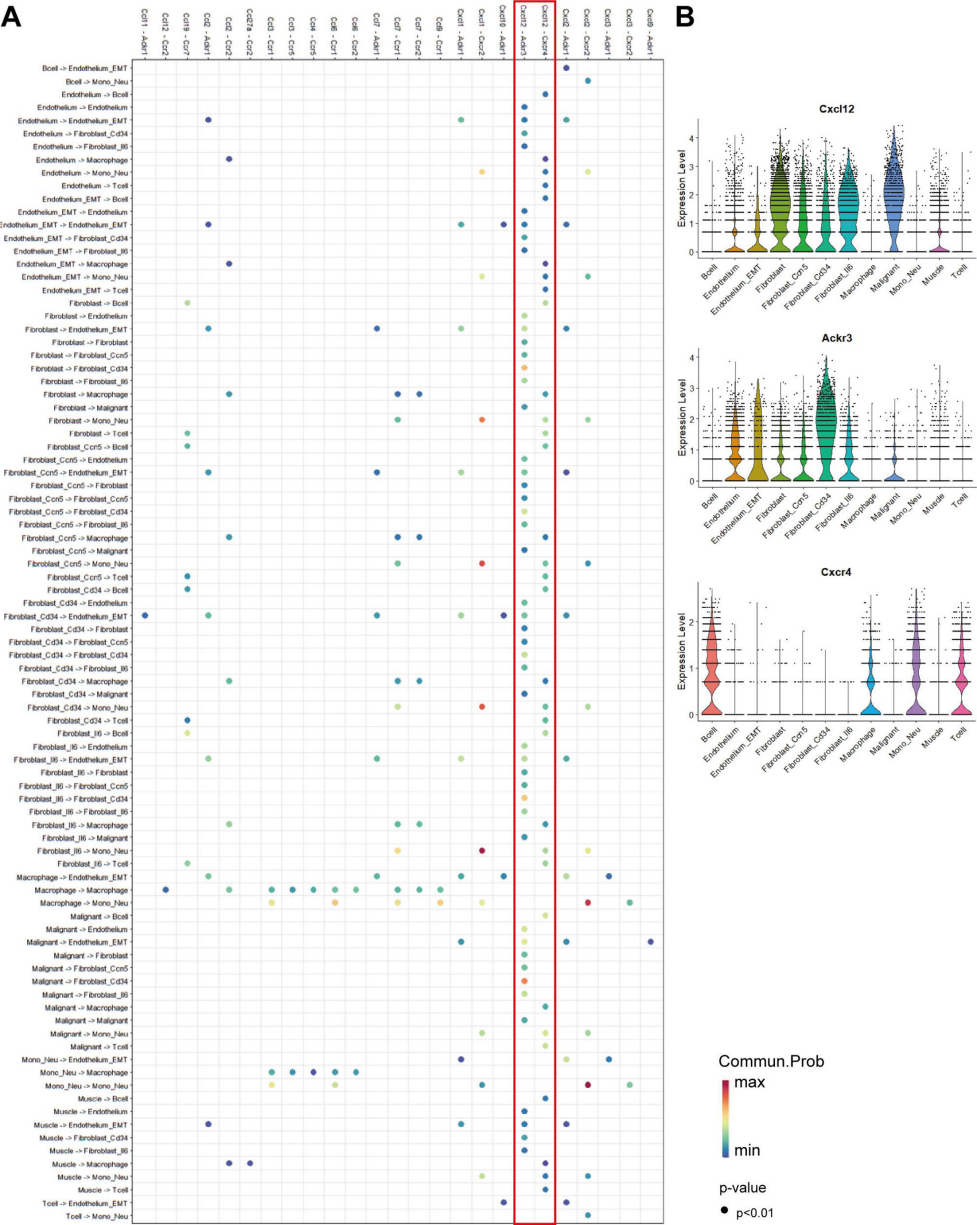
Dot plot illustrating selected ligand–receptor interactions in various cell subsets. (**A**) Ligand and receptor pairs are displayed on the x-axis, while the cell subsets are presented on the y-axis. The color of the dot indicates the signaling probability scores of the receptor–ligand pairs showing a significant interaction among these communicating cell subsets. The *P* values were generated by CellphoneDB, which utilizes a one-sided permutation test to calculate significant interactions. The dots displayed represent interactions with a *P* value <0.01. The communication probability (Commun.Prob) is displayed in the color key. (**B**) The expression distribution of signaling genes *Cxcl12, Ackr3,* and *Cxcr4*, which are involved in the inferred signaling network from the NC, no-AAA, and AAA groups, is visualized through feature plots.

To visualize the intercellular communication network, Figure 6 illustrates the number and strength of interactions between 12 cell clusters. All four types of fibroblast clusters are strongly associated with mono_neu and macrophages. Additionally, fibroblast_Ccn5 is strongly linked to the muscle cluster, and fibroblast_Cd34 is strongly connected to the endothelium_EMT cluster. By analyzing cell communication with CellphoneDB, we identified specific ligand–receptor interaction pairs, such as Cxcl12–Cxcr4 and Cxcl12–Ackr3, which consistently occurred between different clusters (Figure 7A). *Cxcr4* expression was significantly increased in the cluster of immune cells, including macrophages, monocytes, and T and B lymphocytes, while *Ackr3* expression was significantly increased in the cluster of non-immune cells (Figure 7B). By immunostaining, we confirmed the increased expression of Cxcr4 mostly in the aneurysm of the AAA group (Supplementary Figure S2A) and increased expression of Ackr3 mostly in the vascular wall and adventitia of the AAA group (Supplementary Figure S2B).

As a highly conserved seven transmembrane regions protein, Cxcl12 is the only ligand for Cxcr4, which induces the activation of PI3K–Akt signaling pathway and regulates the phosphorylation of ERK1/2 to activate NF–κB and mTOR signaling pathways, thereby regulating cell growth and proliferation [44]. Cxcr4 plays a role in leukocyte chemotaxis during inflammatory conditions in various autoimmune diseases, and elevated levels of Cxcr4 have been observed in various types of human cancers [45]. In our study, we found ubiquitous expression of *Cxcr4* in macrophages, monocytes, and T and B lymphocytes, which might be recruited by Cxcl12 signaling and promote the progression of inflammation, which play a progressive role in the AAA group. Meanwhile, Ackr3 has 10 times more binding affinity for Cxcl12 than the classical receptor Cxcr4. The primary role of Ackr3 is to internalize and deliver its ligand Cxcl12 for lysosomal degradation, thus negatively regulating the Cxcl12-Cxcr4 signaling cascade [46]. The Cxcl12–Ackr3 activates MAPK/ERK signaling pathway to regulate cell survival, migration and differentiation [47]. The cell–cell interaction via Cxcl12–Ackr3 was identified between all clusters of endothelium and fibroblasts by the CellPhone database, which may play a protective role in AAA formation. The differentially distributional expression of Cxcr4 and Ackr3 as shown in Supplementary Figure S2 may imply their antergic roles in different cell types during AAA formation. For example, the higher expression of Cxcr4 and lower expression of Ackr3 in the aneurysm may contribute to the progression of AAA. Thus, the Cxcl12–Cxcr4/Ackr3 axis may play pivotal roles in the initiation, development, and progression of aneurysm.

## Discussion

In this study, we investigated the incidence of AAA in normolipidemic C57BL/6 J mice at different ages. We found that at the age of 20 weeks and 32 weeks with an Ang II concentration of 1.44 mg/kg/day, the incidence of AAA reached 18.2% and 57.1%, respectively. This suggests that the development of AAA is age-dependent. We also found that the diameters of the suprarenal aortic lumen slightly increased with a dose effect of Ang II at the age of 14 weeks. Compared with the apoE^-/-^ mice, the incidence of AAA in the normolipidemic mice is relatively lower. Therefore, the C57BL/6 J mouse model would be more appropriate for testing induction factors rather than protection factors. As advancing age is a risk factor for AAA development in humans [48], which affecting up to 9% of adults older than 65 years of age [49], the incidence of Ang II-induced AAA in mice increased with age, as demonstrated in this study. Therefore, aged C57BL/6 J mice with AAA are better to mimic human AAA.

In human, the most accessible AAA tissue is from advanced lesions that have been dissected during open surgical repair, which cannot provide sufficient evidence of the molecular events at earlier stages. We investigated the cellular landscape of the no-AAA group to mimic the earlier stages of AAA. We found that fibroblasts were the most abundant cell type in the no-AAA tissues. We speculated that the expansion of fibroblasts may be a compensatory reaction to protect the mice from the development of aneurysm. It has been reported that the protective effect may be achieved through modulating extracellular matrix production, thereby enhancing aortic stability [50]. Hong et al. also reported that enhancing collagen synthesis, which increases aortic stiffness, can be a promising approach for treating AAA [51], but further studies are needed to evaluate the benefits and side-effects. The reduction in fibroblasts may be related to the formation of AAA. In addition, C57BL/6 J mice are widely used in studying AAA. We have depicted the difference between the no-AAA and AAA groups induced by Ang II. The cellular landscape can be used to assess the impact of genes or chemicals on alleviating or exacerbating the AAA condition in this mouse models.

The two types of vascular diseases, atherosclerosis and AAA, are strongly interrelated and associated [52]. Although these two diseases develop through different pathogenic mechanisms, they are both closely associated with endothelial dysfunction [20]. The earliest changes that eventually result in the formation of atherosclerotic lesions occur in the endothelium [53]. Endothelium can first sense excessive hemodynamic stimulation, then release and activate various vasoactive substances, disrupt the dynamic balance between proliferation and apoptosis of various cells including ECs, SMCs and fibroblasts, and ultimately alter the morphology, structure, and function of blood vessels. In our study, we identified a unique cluster of malignant arisen after Ang II infusion, and these cells are implicated in lipid uptake. Consistent with previous studies, our results suggest that dyslipidemia and disordered lipid metabolism may be a significant risk factor for AAA development.

The Cxcl12–Cxcr4/Ackr3 axis is involved in both proatherogenic and atherogenesis-protective functions in various cell types [46]. We found that this axis played a role in cell–cell interactions, which is confirmed by the increased expression of Cxcr4 in the aneurysm and increased expression of Ackr3 in the vascular wall and adventitia. Similarly, He et al. found that VSMCs interacted with neutrophils and fibroblasts via Cxcl12–Cxcr4/Ackr3 axis, thereby deteriorating the progression of acute thoracic aortic dissection [54]. We speculate that Cxcl12–Ackr3 plays a protective role, while Cxcl12–Cxcr4 plays a promoting role in AAA formation. Due to the functional heterogeneity and cross-talk between Cxcr4 and Ackr3 at the receptor level and downstream pathways, it will be interesting to explore the clinical implications and modulators of Cxcl12–Cxcr4/Ackr3.

The developmental origin, anatomical location, and other factors contribute to aortic heterogeneity in a physiological state. In recent years, several studies utilizing scRNAseq have focused on the changes in cell clusters within AAA tissues, consistently revealing the heterogenous nature of aortic cells [55]. Chen et al. reported that the reprogramming of normal SMCs into a mesenchymal stem cell-like state led to the formation of aortic aneurysms [56]. Therefore, it has been revealed that various cell types contribute to the formation of AAA, and the roles of different vascular and immune cells in AAA pathogenesis require further characterization. However, since the transcriptional changes observed in this study are based on descriptive analyses, the changes in immune cells may reflect the consequences of the inflammatory response following aortic wall disruption rather than the causes of AAA initiation or progression.

In summary, we have characterized the cellular landscape of the NC, no-AAA, and AAA group. We speculated that upon Ang II infusion, ECs first sense excessive hemodynamic stimulation, then subsequently releasing and activating various vasoactive substances. The dynamic balance may be disrupted between proliferation and apoptosis of various cells including ECs, SMCs, fibroblasts, and immune cells and ultimately to alter the morphology, structure, and function of blood vessels. Although mice were subjected to the same infusion of Ang II, there were interindividual differences in endothelial shear stress and blood flow. This resulted in two distinct groups (no-AAA and AAA) despite receiving the same treatment. Under the influence of Ang II, clusters of endothelium_EMT and malignant EC may appear, which contributes to endothelial dysfunction and triggers a series of cascades in the immune response, leading to SMC apoptosis, monocyte recruitment, and macrophage accumulation. This provides new clues for the intervention and prevention of AAA formation.

## Supplementary material

Online supplementary figure S1

Online supplementary figure S2

## Data Availability

The raw sequence data reported in this paper have been deposited in the Genome Sequence Archive (Genomics, Proteomics & Bioinformatics 2021) in National Genomics Data Center (Nucleic Acids Res 2022), China National Center for Bioinformation/Beijing Institute of Genomics, Chinese Academy of Sciences (GSA: CRA012412) that are publicly accessible at https://ngdc.cncb.ac.cn/gsa/s/mv05GM5r [57, 58].

## Abbreviations

AAA: abdominal aortic aneurysm
Ang II: angiotensin II
BAPN: β-aminopropionitrile monofumarate
ECs: endothelial cells
NC: negative control
SMCs: smooth muscle cells
scRNAseq: single-cell RNA sequencing
