# Supplementary figures and images for: Single-cell RNAseq of Angiotensin II-induced abdominal aortic tissue identifies aneurysm-associated cell clusters in C57BL/6J mice
Source: Biosci Rep. 2025 May 28;45(5):343–60. doi: 10.1042/BSR20241235 (PMC12203967; doi:10.1042/BSR20241235)

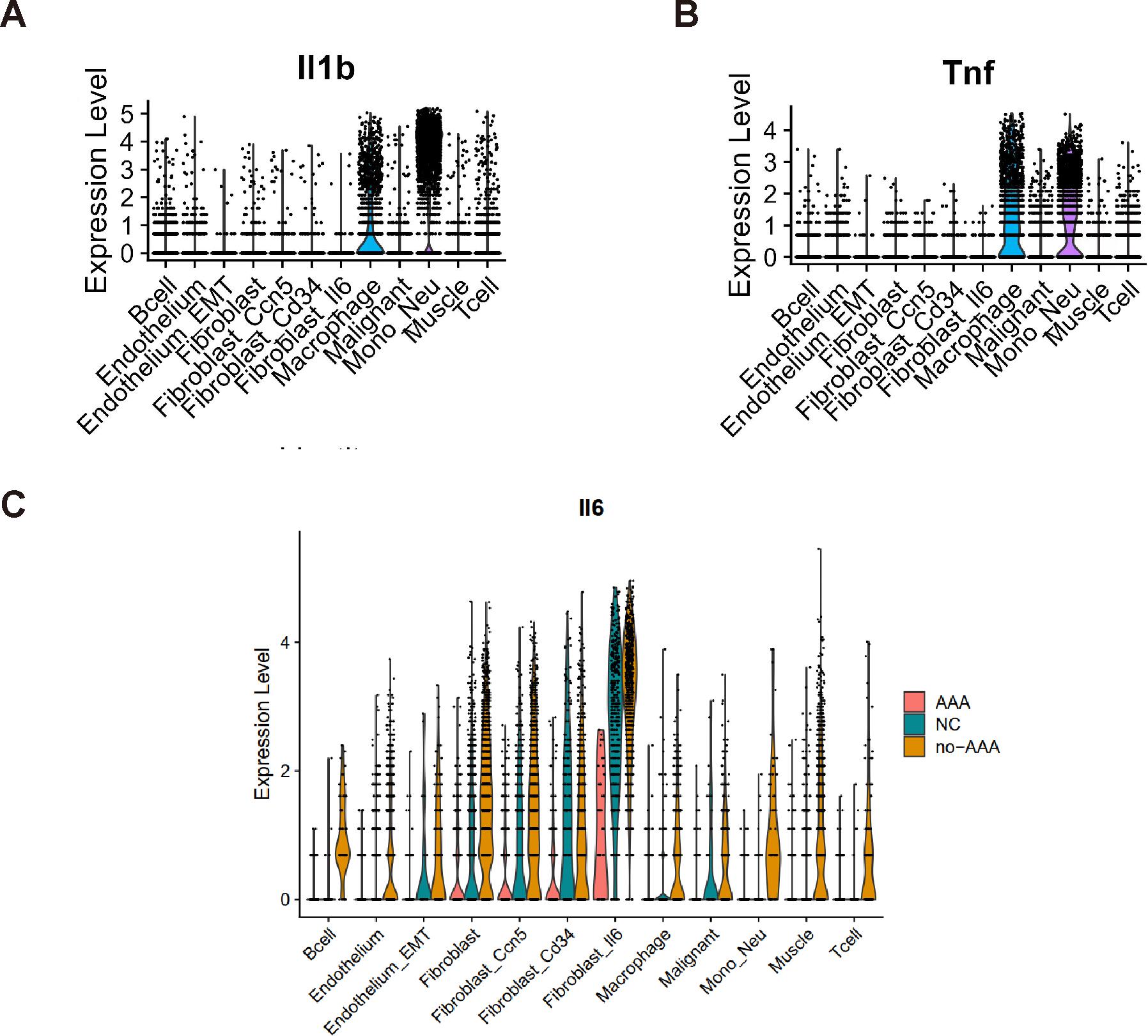

Supplement: Online supplementary figure S1 [file BSR-45-05-BSR20241235-s001.jpg]

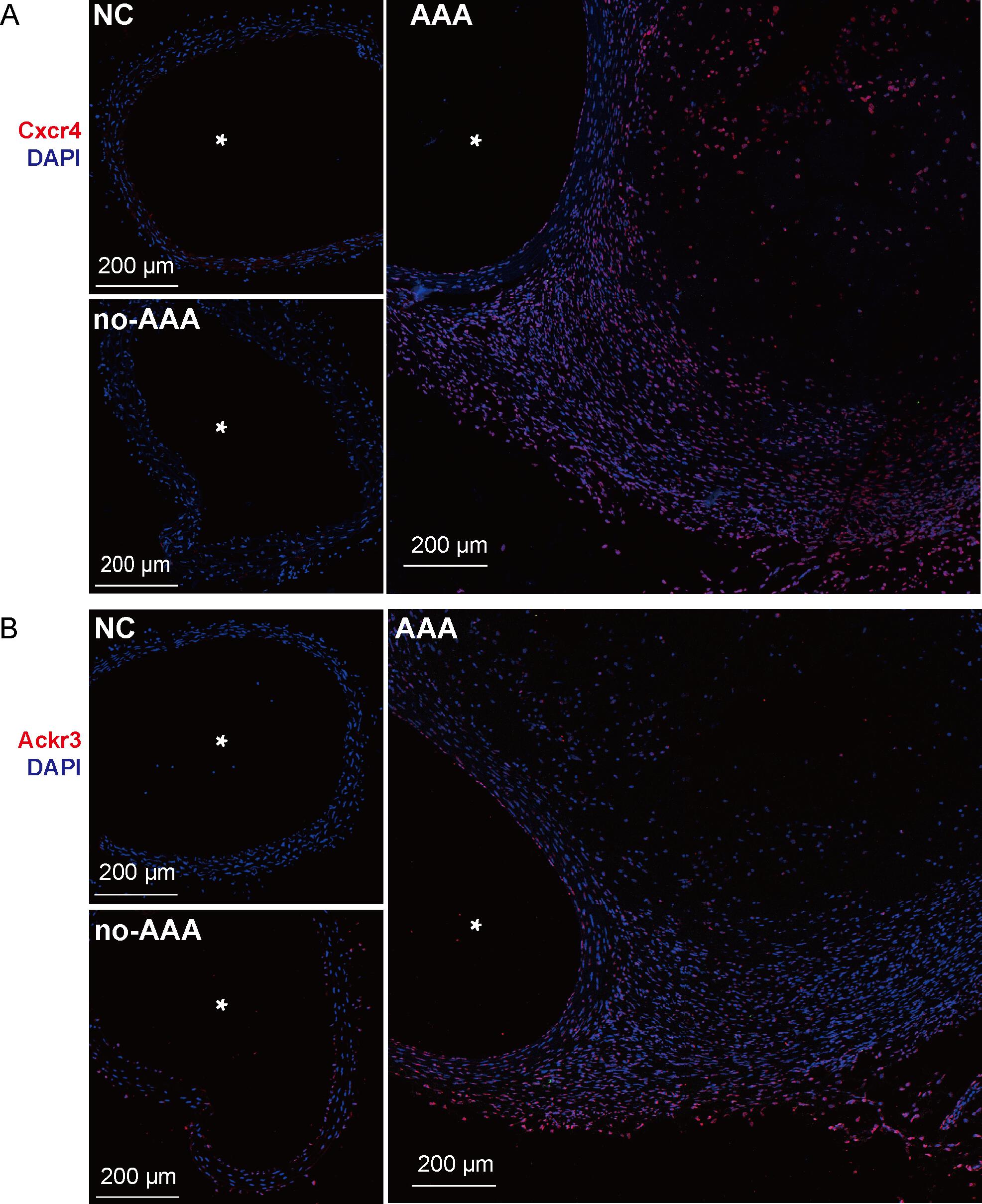

Supplement: Online supplementary figure S2 [file BSR-45-05-BSR20241235-s002.jpg]
